# Disease-specific alterations in central fear network engagement during acquisition and extinction of conditioned interoceptive fear in inflammatory bowel disease

**DOI:** 10.1038/s41380-024-02612-7

**Published:** 2024-05-27

**Authors:** Laura R. Lanters, Hanna Öhlmann, Jost Langhorst, Nina Theysohn, Harald Engler, Adriane Icenhour, Sigrid Elsenbruch

**Affiliations:** 1 Department of Neurology, Center for Translational Neuro- and Behavioral Sciences, University Hospital Essen, University of Duisburg-Essen, Essen, Germany; 2 Department of Medical Psychology and Medical Sociology, Ruhr University Bochum, Bochum, Germany; 3 Department for Internal and Integrative Medicine, Sozialstiftung Bamberg, Bamberg, Germany; 4 Department for Integrative Medicine, Medical Faculty, University of Duisburg-Essen, Essen, Germany; 5 Institute of Diagnostic and Interventional Radiology and Neuroradiology, University Hospital Essen, University of Duisburg-Essen, Essen, Germany; 6 Institute of Medical Psychology and Behavioral Immunobiology, Center for Translational Neuro- and Behavioral Sciences, University Hospital Essen, University of Duisburg-Essen, Essen, Germany

**Keywords:** Neuroscience, Psychology, Psychiatric disorders

## Abstract

Interoceptive fear, which is shaped by associative threat learning and memory processes, plays a central role in abnormal interoception and psychiatric comorbidity in conditions of the gut-brain axis. Although animal and human studies support that acute inflammation induces brain alterations in the central fear network, mechanistic knowledge in patients with chronic inflammatory conditions remains sparse. We implemented a translational fear conditioning paradigm to elucidate central fear network reactivity in patients with quiescent inflammatory bowel disease (IBD), compared to patients with irritable bowel syndrome (IBS) and healthy controls (HC). Using functional magnetic resonance imaging, conditioned differential neural responses within regions of the fear network were analyzed during acquisition and extinction learning. In contrast to HC and IBS, IBD patients demonstrated distinctly altered engagement of key regions of the central fear network, including amygdala and hippocampus, during differential interoceptive fear learning, with more pronounced responses to conditioned safety relative to pain-predictive cues. Aberrant hippocampal responses correlated with chronic stress exclusively in IBD. During extinction, differential engagement was observed in IBD compared to IBS patients within amygdala, ventral anterior insula, and thalamus. No group differences were found in changes of cue valence as a behavioral measure of fear acquisition and extinction. Together, the disease-specific alterations in neural responses during interoceptive fear conditioning in quiescent IBD suggest persisting effects of recurring intestinal inflammation on central fear network reactivity. Given the crucial role of interoceptive fear in abnormal interoception, these findings point towards inflammation-related brain alterations as one trajectory to bodily symptom chronicity and psychiatric comorbidity. Patients with inflammatory conditions of the gut-brain axis may benefit from tailored treatment approaches targeting maladaptive interoceptive fear.

## Introduction

Unravelling the impact of inflammation on the brain mechanisms underlying abnormal interoception has transdiagnostic implications for overlapping clinical conditions involving the gut-brain axis, depressed mood, anxiety, and pain. Translational research accomplished in the context of inflammation-associated depression has provided valuable insight into how acute inflammatory states alter the healthy brain’s responses to signals from inside the body [1–3], as a putative trajectory to psychiatric comorbidity and bodily symptom chronicity. Brain imaging studies in experimental models of acute inflammation support a key role of corticolimbic regions of the extended central fear network, including the amygdala, hippocampus, insula, striatum, and prefrontal cortex [4–8]. Activity and connectivity within this network is demonstrably crucial to the regulation of emotional arousal and fear responses, selective attention, and threat-related memory formation [9], which are all relevant to conditions of the gut-brain axis characterized by chronic abdominal pain in concert with affective comorbidities [10–12]. In our experimental research into fear learning and memory processes underlying abnormal interoception, we recently showed that acute inflammation alters fear network reactivity to conditioned interoceptive threat predictors [4, 8], with effects amplified by depressed mood [4] as a potential vulnerability factor contributing to altered perception and interpretation of bodily signals. These findings in healthy individuals call for studies in patients with chronic inflammatory conditions of the gut-brain axis as a basis for improved mechanistic knowledge and ultimately treatment.

Inflammatory bowel disease (IBD) is a relapsing and remitting chronic inflammatory disease that is associated with pain and mental health comorbidities [13, 14]. In a substantial proportion of IBD patients, these symptoms not only characterize phases of acute disease, i.e., during active bouts of intestinal inflammation, but also phases of clinical remission [15], implicating persisting alterations in interoception. A role of recurring inflammation in persistent symptoms in quiescent IBD is supported by evidence of long-term changes in fear-, anxiety-, and pain-related responses after the resolution of intestinal inflammation in rodent models of colitis [16–19]. In patients with IBD, there exists accumulating evidence of structural brain changes and altered functional brain connectivity, observed during clinical remission, within multiple corticolimbic regions including the amygdala and hippocampus (e.g. [12, 20–22]). Studies using task-based functional brain imaging in IBD patient cohorts also suggest altered corticolimbic neural response relevant to interoception, pain and related psychiatric symptoms [23, 24]. However, whether the central fear circuitry during interoceptive pain-related fear learning and memory processes is altered in IBD has never been tested, despite the crucial role of interoceptive fear and its underlying neurobiology in symptom chronicity and treatment of chronic abdominal pain in irritable bowel syndrome (IBS) as a functional disorder of the gut-brain axis [10, 11, 25, 26].

Building on our earlier interoceptive fear conditioning research in IBS [27], and recent studies on the effects of acute inflammation on interoceptive fear in healthy volunteers [4, 8], we herein present the first functional magnetic resonance imaging (fMRI) study on the neural mechanisms underlying interoceptive fear learning and memory processes in quiescent IBD.

Our aim was to assess central fear network reactivity during interoceptive fear acquisition and extinction in order to test the hypotheses that firstly, IBD patients show greater differential neural responses to conditioned predictors of visceral pain vs. safety in key fear network regions, including amygdala and hippocampus, when compared to healthy controls; and that secondly, alterations within central fear circuitry are specific to IBD as chronic visceral pain condition with underlying relapsing-remitting inflammatory pathology when compared to IBS as a functional visceral pain condition. To this end, a female patient cohort of ulcerative colitis (UC) in remission, women with IBS, and healthy women underwent an established translational fear conditioning paradigm [28]. We focused analyses on comparing groups with respect to differential neural activation within regions of the extended fear network induced by conditioned stimuli (CS^+^) paired with painful rectal distensions as clinically relevant interoceptive unconditioned stimuli (US) versus CS that remained unpaired (CS^−^). Supplemental analyses explored the effects of medications as well as of psychological comorbidity and assessed if group differences in fear network reactivity were specific for the interoceptive (vs. the exteroceptive) pain modality.

## Material and methods

### Participants

A total of 70 female adult volunteers (20 UC, 25 IBS, 25 HC) within an age range of 18–65 years and a body mass index (BMI) >18 and <30 kg/m^2^ were recruited for a comprehensive pain study [23, 28], which included the pain-related fear conditioning paradigm reported on herein. For general exclusion criteria, see S1.1. For patients, a confirmed diagnosis of UC or IBS, respectively was required for inclusion. While patients reporting severe psychiatric comorbidities (e.g., schizophrenia/psychosis, substance abuse, posttraumatic stress disorder) were excluded, mild anxiety or depression symptoms on the Hospital Anxiety and Depression Scale (HADS) as a validated screening questionnaire (see below) were not exclusionary. This is consistent with international recommendations for conducting research studies and clinical trials in conditions of the gut-brain axis [29], as these comorbid psychiatric symptoms are highly prevalent in patient populations with visceral pain and inflammatory conditions involving the gut-brain axis.

The UC cohort consisted only of patients in clinical remission, based on fecal calprotectin levels below 150 μg/g, indicating quiescent intestinal inflammation [30]. In addition, a symptom-based version of the Clinical Activity Index (CAI; [31]) was used, excluding one item concerning laboratory results (i.e., erythrocyte sedimentation rate and hemoglobin). Only patients with symptom-based CAI scores ≤4 (i.e., clear remission) were enrolled, effectively minimizing study-related risks posed by active gut inflammation and avoiding effects of acute inflammation and/or of systemic anti-inflammatory drug treatment (e.g., systemic glucocorticoids) indicated for the treatment of active disease. Accordingly, treatment with systemic glucocorticoids within 4 weeks prior to the study was exclusionary. Drugs regularly prescribed for maintaining disease remission, such as aminosalicylates [32], were not exclusionary (and were also not discontinued) given current treatment guidelines for their use for sustaining clinical remission [33]. Importantly, current evidence supports the action of aminosalicylates to be related to local (i.e., topical) effects on the gastrointestinal mucosa rather than a systemic anti-inflammatory effect [34]. Patients in the IBS cohort fulfilled ROME IV criteria [35], and presented with all stool patterns, i.e., diarrhea-predominant, constipation-predominant, and mixed IBS types. HC were carefully screened for any somatic or mental health conditions (questionnaires for screening of anxiety, depression and gastrointestinal (GI) symptoms, see below) and regular medication use (except hormonal contraceptives and thyroid medication) based on self-report. Of note, the healthy sample herein was age-matched to the UC and IBS patient cohorts from a larger healthy cohort that contributed data to an earlier report [28]. Work was conducted in accordance with the Declaration of Helsinki, and the study was approved by the local Ethics Committee of the University Hospital Essen (protocol number 10-4493). All volunteers gave written informed consent and received monetary compensation for participation.

### Questionnaires

The HADS was used to quantify symptoms of anxiety and depression, with published cut-offs of ≥ 8 (mild/subclinical) and ≥ 11 (clinical) for each subscale, identifying doubtful and potential cases, respectively, of clinically relevant anxiety or depression [36]. In HC, scores above 8 led to exclusion from the study, consistent with standardized criteria for recruitment of healthy volunteers across all our experimental studies based on recommendations in the pain field [37]. In addition to the two subscale scores, we computed total scores for the purposes of sample characterization and analyses of covariance. As a validated and suitable indicator of overall psychological distress, the HADS total score can range from 0 to 42, with higher scores indicating higher levels of overall psychological distress. Frequency of GI symptoms within the past three months was assessed using an established questionnaire, with frequent symptoms above a previously established cut-off (≥11) resulting in exclusion of HC [38]. Current pain severity and interference with daily functioning were measured using the Brief Pain Inventory (BPI, [39]). Chronic perceived stress was assessed with the screening scale of the Trier Inventory for Chronic Stress (TICS, [40]).

### Procedures

**Fig. 1:**
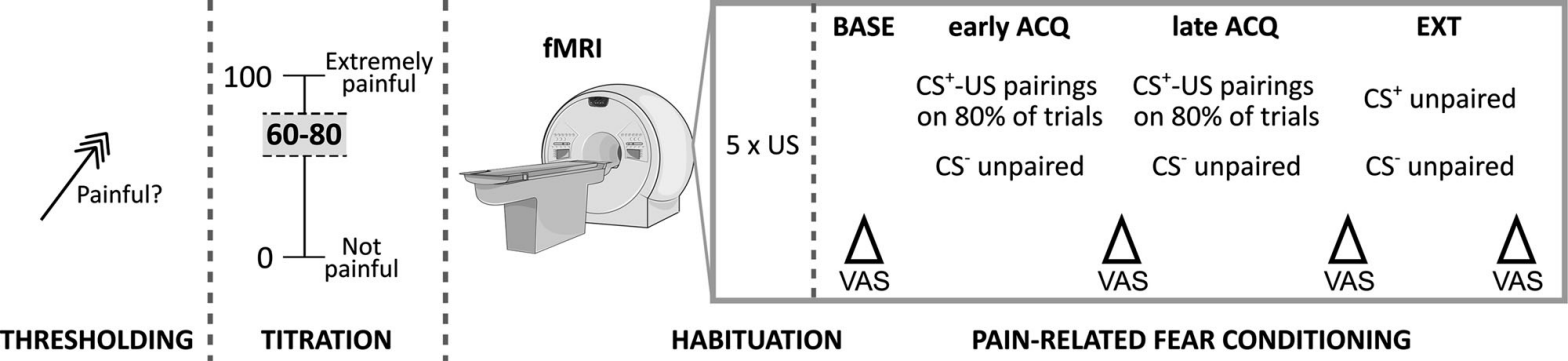
Overview of experimental procedures prior to and during functional magnetic resonance imaging (fMRI). After assessment of pain threshold, titration and matching of individual stimulus intensities to the target range of 60–80 mm (perceived pain intensity on visual analog scale, VAS) was accomplished. Then, functional brain images were acquired in a pain habituation phase, and stimulus intensities were re-adjusted if VAS indicated a deviation from target range. In the pain-related fear conditioning paradigm, blood oxygen level dependent (BOLD) responses to distinct visual conditioned stimuli (CS^+^) paired with either painful rectal distensions or thermal cutaneous pain as unconditioned stimuli (US) and to unpaired cues (CS^−^) were recorded in the acquisition phase (ACQ). In the extinction phase (EXT), only CS^+^ and CS^−^ were presented. CS and US ratings were accomplished at baseline (BASE), mid-acquisition (early ACQ) and after completion of the acquisition phase (late ACQ). The extinction phase was concluded with VAS ratings of CS^+^ and CS^−^.

The multiple threat fear conditioning paradigm has previously been established in healthy volunteers [28]. In this first report on its implementation in two patient cohorts and healthy controls, we focus on conditioned interoceptive fear of visceral pain as US, applied using pressure-controlled rectal balloon distensions (delivered with a barostat system, modified ISOBAR 3 device, G & J Electronics, Toronto, ON, Canada). Besides interoceptive (i.e., visceral) pain stimuli, the paradigm also comprised exteroceptive (i.e., somatic) pain stimuli as US (details in S1.2). As illustrated in Fig. 1, prior to fear conditioning the assessment of pain thresholds, the titration and matching of individual pain stimulation intensities, and a pain habituation phase were accomplished (details in S1.2 and in [23]). Critically, US stimulus intensities were individually titrated to achieve perceived pain intensities within a predefined target range, effectively controlling for interindividual variability in pain sensitivity and minimizing possible effects of condition-specific hypersensitivity (e.g., as has been observed in IBS or in some IBD patients; [11, 41, 42]).

The differential fear conditioning paradigm consisted of acquisition and extinction phases (Fig. 1). In the acquisition phase, one distinct visual CS^+^_VISC_ was contingently paired with visceral pain as US_VISC_ while another visual CS^−^ remained unpaired. The paradigm also contained distinct visual cues (CS^+^_SOM_) paired with somatic pain (US_SOM_). Each CS was presented 10 times, with 8 CS^+^-US pairings (i.e., 80% reinforcement schedule, chosen to induce uncertainty and ensuring robust conditioned responses; [43–45]). CS^+^ were presented 6–12 s before US, with CS^+^ and US co-terminating (i.e., delay conditioning). All US durations were 20 s. In the extinction phase, all CS presentations were accomplished without any US, with a total of five CS per type, again in pseudorandomized order. In both phases, inter-stimulus intervals consisted of a white frame presented on a black screen with a duration of 5–8 s.

As behavioral indicators of conditioned fear, changes in CS valence were acquired with VAS (ends labelled “very pleasant” and “very unpleasant”; “neutral” indicated in the middle of the scale), along with CS-US contingency awareness (i.e., the perceived frequency of a US following a specific CS, VAS ends labelled “never” (0) and “always” (100)). Additionally, US intensity (and unpleasantness, not reported herein) were assessed immediately before (baseline), mid-acquisition (early ACQ) and after the acquisition phase (late ACQ) using VAS (see Fig. 1). All visual stimuli and online rating scales were presented with Presentation® software (Neurobehavioral Systems, Albany, CA, USA) projected onto a mirror mounted on the MR head coil, and digitized VAS ratings were accomplished with an MRI-compatible hand-held fiber optic response device (LUMItouchTM, Photon Control Inc., Burnaby, BC, Canada).

### Brain imaging data acquisition and analyses

All MR images were acquired using a whole-body 3 Tesla scanner (Skyra, Siemens Healthcare, Erlangen, Germany) equipped with a 32-channel head coil. Established acquisition protocols and preprocessing steps were applied, as previously published for T1-weighted structural MR-images used for a larger voxel-based morphometry analysis [46] and blood-oxygen-level-dependent (BOLD) data of pain-induced responses in the habituation phase [23] in the same cohorts (for expanded methods, see S1.3). First-level analyses were performed using a general linear model applied to the EPI images with experimental phases modelled as separate sessions. The time series of each voxel was fitted with a corresponding task regressor that modelled a box car convolved with a canonical hemodynamic response function (HRF). As regressors of interest, CS^+^_VISC_, CS^−^ and US_VISC_ were included. Additionally, CS^+^_SOM_ and US_SOM_ were entered as nuisance regressors. All CS were modelled with variable durations between 6 and 12 s, i.e., until US onset). The six realignment parameters for translation and rotation were further implemented as multiple regressors for motion correction. After model estimation, first-level contrasts and respective reverse contrasts were computed for CS^+^_VISC_ > CS^−^ for the acquisition and extinction phases, respectively. As explained above, VAS ratings were acquired mid-ACQ and after ACQ, which is why these contrasts were computed for the early (i.e., first half) and for the late (i.e., second half) of the acquisition phase, in line with our earlier studies (e.g., [8]). For US_VISC_-related analyses, first-level contrasts were US_VISC_ > Rest, computed only for early and late acquisition phases since no US were delivered during extinction. To test the hypothesized group differences, two-sided independent samples t-tests were accomplished on the second level for each phase, comparing UC and HC (i.e., UC>/<HC) and UC and IBS (i.e., UC>/<IBS). As part of supplemental analyses, group comparisons were accomplished after the exclusion of UC patients treated with systemically acting anti-inflammatory drugs (see S2.1); group comparisons with HADS total score as an additional covariate of no interest to explore the contribution of overall psychological distress to group differences (see S2.2); and focused analyses of differential neural activation during exteroceptive fear acquisition and extinction, respectively, within those brain regions in which group differences were found for interoceptive fear learning to explore specificity to US modality (see S2.3).

All analyses focused on a priori defined regions of interest (ROI) of the fear and extinction networks [47–49], including amygdala, hippocampus, ventromedial prefrontal cortex (vmPFC), anterior insula (aINS), anterior cingulate cortex (ACC), thalamus, and basal ganglia (putamen, pallidum, caudate) (for details on segmentation, masks and plots, see S2.1). For all reported ROI analyses, familywise-error (FWE) correction for multiple testing was used with statistical significance set at *p*_FWE_ < 0.05 and a minimal cluster size (*k*_*E*_) of 3, and coordinates refer to the MNI space. Parameter estimates from peak-voxels identified in significant group comparisons were extracted from the respective ROI to visualize the direction of observed effects, and to accomplish exploratory correlational analyses.

### Statistical analyses of behavioral data

Statistical analyses were performed using IBM SPSS Statistics 27 (IBM Corporation, Armonk, NY, USA). Baseline group characteristics were compared by two-sided independent samples *t*-tests. Equivalent to analyses of differential neural responses to CS_VISC_, we computed differential cue valence (ΔCS_VISC_) as individual delta (Δ) scores for the CS^+^_VISC_ relative to the CS^−^ for each learning phase, to ascertain successful fear learning and to evaluate possible group differences in the magnitude of learning. Hence, aiming to primarily test for *group × time* interaction effects, changes in differential cue valence during acquisition and extinction were analyzed by repeated-measures analysis of variance (ANOVA) or analyses of covariance (ANCOVA, see S2.2). These were accomplished for each learning phase with *time* as within-subject factor and *group* as between-subject factor. US_VISC_ pain intensity ratings were analyzed using ANOVA with the same factors. Contingency awareness of CS^+^_VISC_-US_VISC_ association was compared across groups using two-sided independent samples *t*-tests.

In all AN(C)OVA, Greenhouse-Geisser correction was applied, when the assumption of sphericity was violated. *Post-hoc* comparisons between groups were performed using two-sided independent samples *t*-tests. Effect sizes are reported as partial eta squared (*η*_*p*_^*2*^) for AN(C)OVA and as Cohen’s *d* for *t*-tests. Exploratory correlational analyses were accomplished using Pearson’s *r*. The alpha level for accepting statistical significance was set at *p* < 0.05. Results are reported as mean ± standard error of the mean (*SEM*) unless indicated otherwise.

## Results

### Sample characteristics

The final sample consisted of 20 UC patients, 25 HC, and 23 IBS patients, as data from 2 patients with IBS were excluded due to technical difficulties. All UC patients were clearly in remission, as supported by CAI scores ≤ 4 (0.60 ± 0.18; range: 0-3) and fecal calprotectin concentrations <150 μg/g (37.22 ± 5.22 μg/g; range: 7.99–105.30 μg/g), in line with strict exclusion criteria regarding current disease activity. Rectal pain thresholds in UC patients (34.00 ± 10.46 mmHg) did not significantly differ from HC (40.00 ± 12.00 mmHg; *p* = 0.085) or from IBS patients (37.83 ± 10.09 mmHg; *p* > 0.1). Fifteen UC patients (75%) were taking colitis-related medications, the majority consisting of locally acting anti-inflammatory drugs (i.e., aminosalicylates, *N* = 11; local corticosteroids, *N* = 1). A minority of patients reported other anti-inflammatory drugs (i.e., a TNF-α blocker, *N* = 2; the immunosuppressant azathioprine, *N* = 2).

As detailed in Table 1, the UC and healthy cohorts were comparable with respect to age and BMI, but UC patients reported significantly more GI symptoms and greater pain severity. Total HADS scores were significantly higher in the UC versus the healthy cohort, indicating greater overall psychological distress, which was attributable to higher symptoms of anxiety but not depression in UC. The UC and healthy cohorts did not differ with respect to chronic perceived stress.

**Table 1:** Sociodemographic, clinical, and psychological characteristics of study cohorts.

	UC (*N* = 20)	HC (*N* = 25)	IBS (*N* = 23)	Group comparison	*t*	*p*	*d*
Age	39.20 ± 2.84	42.04 ± 2.61	46.91 ± 2.28	UC vs. HC	0.34	0.467	0.22
				UC vs. IBS	2.14	0.038*	0.66
BMI	23.03 ± 0.68	23.03 ± 0.56	23.13 ± 0.84	UC vs. HC	0.01	0.996	0.02
				UC vs. IBS	0.09	0.926	0.03
GI symptoms	9.45 ± 1.43	3.68 ± 0.61	15.09 ± 1.02	UC vs. HC	3.71	0.001**	1.20
				UC vs. IBS	3.27	0.002**	1.00
HADS total score	10.45 ± 1.02	6.76 ± 0.86	15.09 ± 1.27	UC vs. HC	2.78	0.008**	0.83
				UC vs. IBS	2.79	0.008**	0.85
HADS anxiety	10% clinical	0% clinical	39% clinical				
	6.95 ± 0.69	3.68 ± 0.50	9.09 ± 0.68	UC vs. HC	3.94	<0.001***	1.18
				UC vs. IBS	2.19	0.034*	0.67
HADS depression	0% clinical	0% clinical	13% clinical				
	3.50 ± 0.54	3.08 ± 0.50	6.00 ± 0.69	UC vs. HC	0.57	0.571	0.17
				UC vs. IBS	2.80	0.008**	0.85
BPI pain severity	5.53 ± 1.82^†^	1.20 ± 0.68	9.48 ± 1.82	UC vs. HC	2.23	0.036*	0.75
				UC vs. IBS	1.52	0.136	0.47
BPI pain interference	8.95 ± 3.61^†^	1.88 ± 1.08	14.04 ± 3.40	UC vs. HC	1.87	0.075	0.64
				UC vs. IBS	1.02	0.312	0.32
TICS chronic stress	20.85 ± 2.08	15.68 ± 2.08	25.17 ± 1.79	UC vs. HC	1.87	0.069	0.56
				UC vs. IBS	1.75	0.089	0.53

Sociodemographic, clinical, and psychological characteristics of study cohorts based on independent sample t-tests comparing patients with ulcerative colitis (UC) with healthy controls (HC) and with irritable bowel syndrome (IBS) as patient comparison group. Data are shown as mean ± standard error of the mean. For HADS anxiety and depression subscales, the percentage of participants (within groups) presenting with clinically relevant scores, i.e., with scores exceeding the published threshold of ≥11, is additionally provided (% clinical). Note that in all groups, pre- and postmenopausal women were represented (UC: 13 premenopausal, 10 using hormonal contraceptives; HC: 15 premenopausal, 6 using hormonal contraceptives; IBS: 12 premenopausal, 3 using hormonal contraceptives).

*BMI* body mass index, *BPI* Brief Pain Inventory, *HADS* Hospital Anxiety and Depression Scale, *TICS* Trier Inventory for Chronic Stress, *UC* ulcerative colitis.

^†^*N* = 19. *p < 0.05, **p < 0.01, ***p < 0.001.

The IBS cohort presented with diarrhea-predominant (*N* = 9), constipation-predominant (*N* = 4), mixed (*N* = 9), and unspecified (*N* = 1) bowel habit disturbances. Four IBS patients (17%) were taking prescription drugs including selective serotonin reuptake inhibitors (*N* = 1), muscarine receptor antagonists (N = 2), and loop diuretics (*N* = 1). Compared to the UC cohort, IBS patients were older, and had higher overall psychological and clinical symptom burden, reflected by higher GI symptoms and greater total HADS as well as HADS anxiety and depression subscale scores (Table 1).

### Behavioral and neural responses to visceral pain as interoceptive US_VISC_

Baseline US_VISC_ intensity ratings were within the intended target range and comparable for all groups (69.35 ± 1.85 mm for UC; 70.88 ± 1.29 mm for HC; 67.17 ± 2.18 mm for IBS, all *p* > 0.1). Over the course of acquisition, no significant group differences in US_VISC_ ratings emerged (all group and group x time interaction effects: *p* > .1). However, a significant main effect of time was observed [*F*(2,130) = 19.94, *p* < .001, *η*_*p*_^2^ = 0.24], with increasing perceived pain intensity of US_VISC_ from baseline to the end of the acquisition phase in all groups (difference from baseline to late ACQ: 7.45 ± 2.52 mm for UC; 4.56 ± 2.45 mm for HC; 13.09 ± 2.47 mm for IBS). At the neural level, ROI-analyses comparing UC vs. HC revealed no US_VISC_-related group differences, except for altered engagement of the right amygdala in early acquisition (*x* = 20, *y* = −2, *z* = −16, *t* = 4.26, *p*_FWE_ = 0.002, *k*_E_ = 17). The comparison UC vs. IBS revealed no significant findings.

### Acquisition of conditioned interoceptive fear responses to CS_VISC_

Analyses of conditioned changes in differential cue valence (i.e., ΔCS_VISC_) revealed a significant main effect of time [*F*(1.62, 105.39) = 32.97, *p* < 0.001, *η*_*p*_^*2*^ = 0.34], but no significant group or group × time interaction effects (all *p* > 0.1). Successful and comparable differential learning in all groups was supported by the expected increase in negative valence of CS^+^_VISC_ relative to CS^−^ (increase in ΔCS_VISC_ from baseline to late ACQ: 73.70 ± 21.04 mm for UC; 54.24 ± 14.52 mm for HC; 70.70 ± 17.54 mm for IBS). This was also reflected by adequate and comparable contingency awareness of the CS^+^_VISC_-US_VISC_ association in all groups (UC: 83.20 ± 4.59%, HC: 73.48 ± 4.93%, IBS: 74.96 ± 4.06%; all *p* > 0.1).

**Fig. 2:**
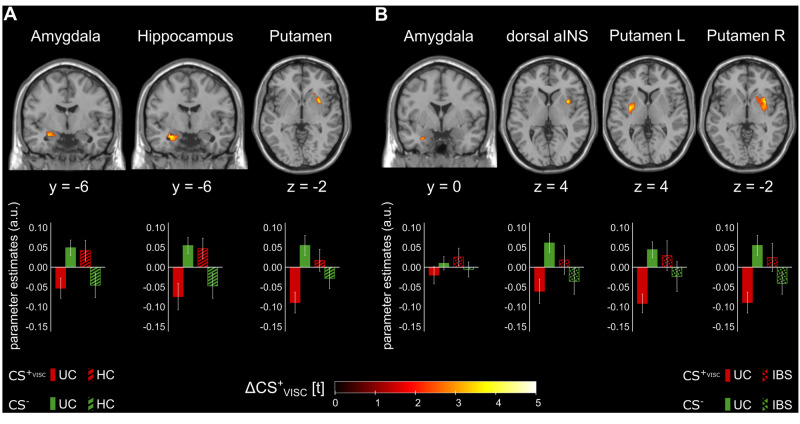
Group differences in differential blood oxygen level dependent (BOLD) responses induced by conditioned predictors of interoceptive pain (i.e., ΔCS_VISC_) in the late acquisition phase in patients with ulcerative colitis (UC) compared to (**A**) healthy controls (HC) and (**B**) patients with irritable bowel syndrome (IBS). For visualization purposes, activations were superimposed on a structural T1-weighted MR-image, thresholded at *p* < 0.01 uncorrected, and masked for the a-priori defined regions of interest. The color bar indicates t-scores (for statistical details, see Table 2), and parameter estimates are provided to indicate the direction of effects. aINS anterior insula, a.u. arbitrary units, CS conditioned stimuli, L left, R right.

At the neural level, BOLD analyses comparing UC and HC revealed altered differential activation induced by conditioned predictors of visceral pain (i.e., ΔCS_VISC_) in UC patients within left amygdala, left hippocampus and right putamen during late acquisition (Fig. 2A; for details, see Table 2, upper section). When the UC cohort was compared to IBS patients, differences also emerged within left amygdala, bilateral putamen, and right dorsal aINS during late acquisition (Fig. 2B; Table 2). While in all regions HC and IBS showed greater CS^+^_VISC_ relative to CS^−^ activation, the pattern of differential neural activation was reversed in UC, with greater activation induced by the CS^−^ relative to the CS^+^_VISC_ (Fig. 2).

**Table 2:** Differential neural responses to conditioned predictors of interoceptive pain during acquisition and extinction phases.

Contrast	ROI	H	MNI coordinates			*t*	*p*_FWE_	*k*_E_
			*x*	*y*	*z*			
ACQ								
UC < HC								
Early ACQ	–	–	–	–	–	–	–	–
Late ACQ	Amygdala	L	−28	−6	−18	3.66	0.011	24
	Hippocampus	L	−26	−6	−20	3.96	0.019	7
	Putamen	R	34	8	−2	4.06	0.015	8
UC < IBS								
Early ACQ	–	–	–	–	–	–	–	–
Late ACQ	Amygdala	L	−28	0	−28	3.49	0.020	3
	dorsal aINS	R	34	12	4	3.76	0.029	6
	Putamen	L	−32	−6	4	3.73	0.041	6
		R	34	8	−2	4.34	0.009	17
EXT								
UC < HC	–	–	–	–	–	–	–	–
UC < IBS	Amygdala	L	−24	−6	−12	3.57	0.018	32
	ventral aINS	L	−38	4	−10	3.83	0.015	6
	Thalamus	R	4	−26	6	4.13	0.016	5

Group differences in differential blood oxygen level dependent (BOLD) responses induced by conditioned interoceptive pain predictors (ΔCS_VISC_: CS^+^_VISC_ > CS^−^) during interoceptive fear acquisition and extinction. Results of voxel-based analyses in region of interest analyses are provided, and exact unilateral *p* values are given (all *p*_FWE_ < 0.05). For supplemental results of an analysis excluding UC treated with TNF-α blocker (*N* = 2) or azathioprine (*N* = 2), see S2.1; for results with HADS total score as covariate of no interest, see S2.2; for results of analyses on exteroceptive pain predictors, see S2.3.

*ACQ* acquisition, *aINS* anterior insula, *CS* conditioned stimulus, *EXT* extinction, *FWE* family-wise error, *H* hemisphere, *HC* healthy controls, *IBS* irritable bowel syndrome, *MNI* Montreal Neurological Institute, *ROI* region of interest, *UC* ulcerative colitis.

Interestingly, correlational analyses revealed a significant association between altered hippocampal engagement in differential responses to visceral threat predictors in UC with chronic stress (*r* = −0.51, *p* = 0.021). Analyses aiming to discern associations between altered CS_VISC_− and US_VISC_-related neural activation within UC over the course of conditioning further revealed a positive correlation between CS^+^_VISC_-induced neural response within hippocampus in the late acquisition phase and US_VISC_-induced neural response within right amygdala in the early acquisition phase (*r* = 0.45, *p* = 0.047).

### Extinction of conditioned interoceptive fear responses to CS^+^_VISC_

During extinction, analyses of conditioned changes in differential cue valence (i.e., ΔCS_VISC_) yielded a significant time effect [*F*(1,65) = 19.14, *p* < 0.001, *η*_*p*_^*2*^ = 0.23], but no significant main effect of group (*p* > 0.05) or group x time interaction (*p* > 0.1), reflecting extinction of conditioned responses in all groups (decrease in ΔCS_VISC_ valence from late ACQ to EXT: −23.70 ± 19.67 mm for UC; −32.80 ± 9.58 mm for HC; −51.48 ± 13.67 mm for IBS).

**Fig. 3:**
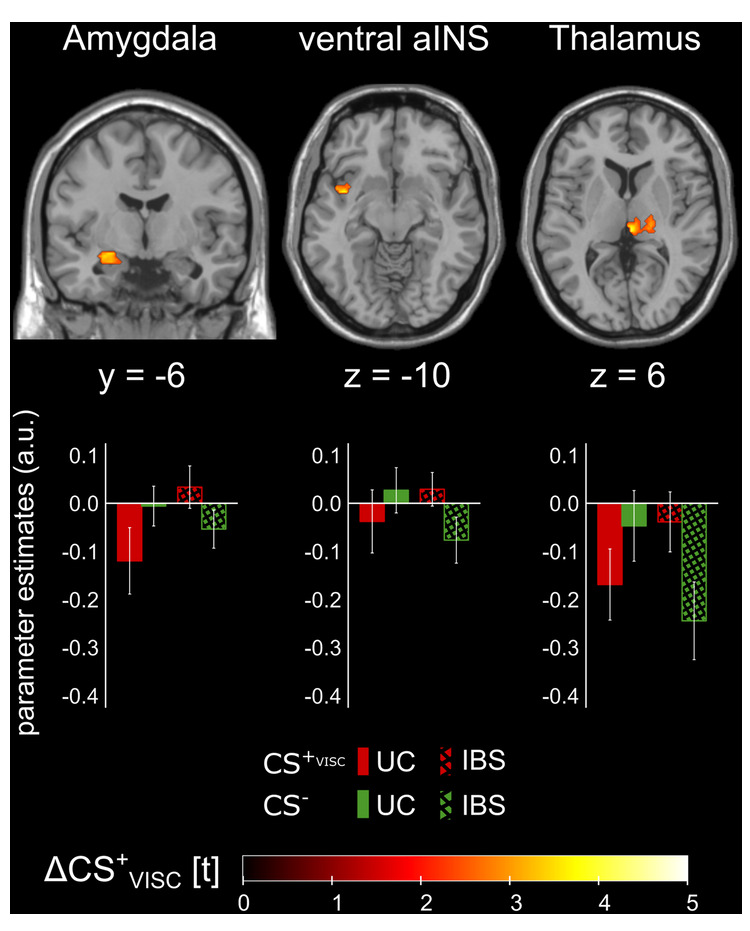
Group differences in differential blood oxygen level dependent (BOLD) responses induced by conditioned interoceptive pain predictors (i.e., ΔCS_VISC_) in the extinction phase in patients with ulcerative colitis (UC) compared to patients with irritable bowel syndrome (IBS). For visualization purposes, activations were superimposed on a structural T1-weighted MR-image, thresholded at *p* < 0.01 uncorrected, and masked for the a-priori defined regions of interest. The color bar indicates t-scores (for statistical details, see Table 2), and parameter estimates are provided to indicate the direction of effects. aINS anterior insula, a.u. arbitrary units, CS conditioned stimulus.

BOLD analyses comparing UC and HC revealed no significant group differences in differential ΔCS_VISC_-induced neural responses. However, when the UC cohort was compared to IBS patients, differential engagement in response to ΔCS_VISC_ was observed within amygdala, ventral aINS, and thalamus (Fig. 3; Table 2, lower section).

### Supplemental analyses

Firstly, given that the UC cohort was not entirely untreated, possible effects of anti-inflammatory medications were explored. As detailed in S2.1, supplemental data in subgroups of UC patients excluding systemically acting drugs and exploring response patterns in completely untreated patients confirmed findings in the full sample. Secondly, in light of group differences observed in HADS scores reflecting psychiatric comorbidity, supplemental ANCOVA with HADS total score as a covariate were accomplished. As detailed in S2.2, behavioral and pain-related results were not appreciably altered. However, clusters reflecting significant group differences in fear network reactivity during acquisition and extinction of interoceptive fear failed to reach suprathreshold levels with the inclusion of the covariate of no interest. Finally, we explored the specificity of group differences reported for interoceptive (i.e., visceral pain-related) fear to pain modality. Results of selected analyses of conditioned exteroceptive fear of somatic pain as US support that altered fear network reactivity is specific to the interoceptive, visceral modality, as explained in S2.3.

## Discussion

We herein report on the first conditioning study assessing central fear network reactivity during interoceptive fear learning and extinction in patients with IBD as a relapsing-remitting chronic inflammatory disease compared not only to HC but also to IBS as a functional visceral pain comparison group. As a key finding, we document disease-specific alterations in central fear network engagement during the acquisition and extinction of interoceptive fear in our cohort of UC patients tested in clinical remission. These results support our assumption that relapsing-remitting bouts of intestinal inflammation have a long-lasting impact on the reactivity of this corticolimbic network to emotionally salient stimuli along the gut-brain axis. This could reflect a mechanism underlying transdiagnostic trajectories to abnormal interoception and psychiatric comorbidities in overlapping conditions involving chronic visceral pain and inflammation.

During fear acquisition, patients with UC demonstrated distinctly altered engagement of key regions of the extended central fear network, including amygdalar and hippocampal subregions, in support of our first hypothesis. As a crucial brain structure in fear conditioning [50], the amygdala is part of a highly interconnected neural network of corticolimbic brain regions regulating emotional arousal, selective attention, and threat-related memory formation [9, 51], which is reportedly sensitive to inflammation and relevant to inflammation-related psychiatric comorbidity [5, 7, 52]. The few other existing task-based fMRI studies in quiescent IBD corroborate our finding of altered reactivity in amygdala and interconnected corticolimbic regions, observed during uncertainty [24], stress [53], and picture-based emotional processing tasks [54]. This converging evidence suggests altered corticolimbic network reactivity to emotionally salient stimuli in quiescent IBD, presumably as a long-term or persisting effect of repeated inflammatory bouts originating in the intestines on brain regions of the extended central fear network. In support of this notion, in animal models of colitis persisting changes in visceral pain-related responses have been demonstrated after the resolution of acute inflammation [16, 17, 19]. In healthy humans undergoing fear acquisition during endotoxin-induced acute inflammation, we recently observed selective engagement of amygdala, hippocampus and other regions during re-exposure to conditioned fear cues after the resolution of inflammation [8]. Interestingly, in this study post-inflammatory effects were only observed on neural responses to interoceptive but not to exteroceptive pain predictors. Consistent with these findings, supplemental analyses herein could not reproduce similar group differences in fear network reactivity for exteroceptive fear of somatic pain as US, suggesting specificity to the interoceptive modality. This is in keeping with the notion that interoceptive, visceral signaling is highly salient and prioritized in the brain’s sensory, attentional, and emotional response systems [8, 28].

Our finding that altered amygdala reactivity during fear acquisition emerged not only in group comparisons of UC vs. HC but also in UC vs. IBS supports the second hypothesis assuming disease-specific fear network alterations. In both reference groups (i.e., HC and IBS), CS-US pairings during fear acquisition induced the typical differential neural activation in fear network regions, comprising greater CS^+^_VISC_ relative to CS^−^-induced responses. Surprisingly, this neural response pattern was reversed in UC patients, with greater activation induced by the CS^−^ relative to the CS^+^_VISC_. Hence, central fear network reactivity in UC was not per se enhanced, as we had expected, but rather altered in a more complex manner. The abnormal directionality of differential neural responding in UC must be interpreted in light of the adaptive nature of threat and safety learning during fear conditioning [28], involving CS-specific changes in attention, reappraisal, and perceptual acuity during the anticipation of threat. Based on this, the adaptive preparation for impending interoceptive threat appears to be compromised in UC, which is not exclusively driven by reactivity to danger (CS^+^_VISC_) but also to safety (CS^−^) cues. Safety processing normally facilitates fear inhibition and adaptive security seeking [55, 56]. The observed reactivity to CS^−^ in UC patients therefore constitutes intriguing novel insight into impaired human safety learning, as a distinct emotional response processing system, in the context of chronic inflammation. This is particularly interesting since mice exhibited fear-related responses in conditioned safety contexts after undergoing repeated bouts of gut inflammation [18], calling for further translational research into mechanisms underlying maladaptive safety-seeking and avoidance behavior in patients with chronic visceral pain [25].

Interrupting the vicious cycle of fear, hypervigilance and avoidance is at the core of cognitive-behavioral treatment approaches built on the principles of extinction learning [25]. The present study offers first insight into extinction learning in patients with a chronic inflammatory condition, complementing evidence in healthy humans suggesting an impact of acute inflammation on interoceptive fear extinction circuitry [8]. Analyses of the extinction phase herein revealed group differences in differential cue-related neural responses in amygdala, anterior insula, and thalamus between the UC and IBS cohorts, yet no differences when compared to HC. In support of our second hypothesis assuming specificity to IBD vs. IBS, the combined results from the acquisition and extinction phases are of interest to the ongoing debate on overlap and differences between IBD and IBS [23, 57–61], expanding on research in other chronic inflammatory pain conditions unrelated to the gut-brain axis [62, 63]. Since IBD and IBS patients share intestinal and psychological symptom phenotypes, the observed patient group differences are likely attributable to their distinct underlying pathophysiology, particularly the effects of repeated bouts of intestinal inflammation on the brain along afferent pathways of the gut-brain axis. However, conclusions about cause-effect relationships would require prospective studies, ideally assessing patients repeatedly during phases of active vs. inactive disease, or cross-sectional studies in well-matched IBD cohorts in different disease phases. An elegant alternative approach was recently accomplished in patients undergoing interferon and anti-TNF therapies, elucidating the mechanistic role of amygdala emotional reactivity in inflammation-associated psychiatric comorbidity [5]. However, issues of feasibility and safety of any such approaches in patients with IBD, especially using paradigms involving rectal distensions as a clinically relevant model of interoceptive pain, as well as limitations arising from treatment requirements with systemically acting anti-inflammatory drugs during exacerbations, remain to be resolved.

Given the high comorbidity of IBD and affective symptoms [14], and our recent experimental data supporting that inflammation and depressed mood interact as vulnerability factors for visceral pain [4], future research should elucidate more vulnerable subgroups of patients, e.g., patients with high levels of chronic stress or affective comorbidities. It is conceivable that the effects observed herein (i.e., in patients in remission with relatively low overall psychological distress) may be even more pronounced in patients with higher disease burden. Enhanced fear network reactivity may precipitate overt behavioral impairments, such as hypervigilance, maladaptive avoidance and hyperalgesia upon re-exposure to pain [8], especially when additional risk factors or triggers, such as acute inflammatory bouts, stressful events, or episodes of depression [4] come into play. This could explain why effects herein were only observed at the neural level, consistent with our earlier findings in healthy volunteers [8, 64]. Given group differences herein in HADS scores, supplemental analyses with overall psychological distress as a covariate were accomplished. While results remained essentially the same for group differences in US-related neural activation, CS-related group differences in fear network reactivity were no longer significant. Hence, even subclinical psychiatric comorbidity appears to contribute to group differences in altered fear network reactivity in IBD, which is not surprising given the intricate connections between chronic inflammation and the brain’s emotional reactivity networks on the one hand, and the well-established role of peripheral inflammation in driving both pain and psychiatric comorbidity on the other hand [1, 3, 6, 65]. Furthermore, intriguing correlational results observed exclusively within the UC cohort underscore disease-specific interactions between pain- and fear-related neural responses that appear to be shaped by psychological factors: Hippocampal responses to CS^+^_VISC_ in the late acquisition phase correlated with aberrant amygdala activation induced by visceral pain (i.e., the US_VISC_) in the early acquisition phase. Since the correlation was negative, greater pain-related amygdala reactivity to visceral pain as an interoceptive threat with pronounced affective components [66–68] may drive impaired (i.e., reduced differential) engagement of hippocampus as a key region for threat-related memory formation. The CS^+^_VISC_-induced aberrant hippocampal activation further correlated with higher perceived chronic stress in UC patients, which is consistent with findings in animal models (e.g., [69]) and intriguing given recent evidence for an association of stress-related alterations of hippocampal functioning and inflammation [69]. In IBD, the broad role of chronic stress as a risk factor for disease exacerbation and psychiatric comorbidity is well-documented [70], further supported by evidence that the physiological stress systems are dysregulated (e.g., [71, 72]). The present correlational results are therefore not only in line with existing knowledge on chronic stress in IBD, but they also suggest that differential interoceptive fear-related memory formation may be impaired as a function of chronic stress, expanding on the notion that learned interoceptive fear shapes “gut memories” [28, 73], with implications for treatment approaches based on the extinction of fear responses.

Together, collective findings from experimental models of acute inflammation in humans, rodent models of chronic inflammation, and data from patient cohorts support the role of chronic inflammation as one trajectory to altered interoceptive fear processing, abnormal interoception, and chronic pain, likely in concert with psychological risk factors such as chronic stress, anxiety and depression that demonstrably impact both on central fear network reactivity and on symptom-related outcomes, including pain reports. In IBD, pain persisting beyond phases of active inflammation afflicts a substantial proportion of patients [15], is largely untreated [74], and has detrimental effects on health-related quality of life [75, 76]. While evidence-based psychological interventions are available for IBS [77], including cognitive-behavioral approaches targeting sustained extinction of fear [25], treatment of IBD is still mainly focused on the induction of remission in patients with active disease [65, 78]. Therefore, unravelling the impact of chronic inflammation, stress and depression on the formation and extinction of interoceptive fear in IBD has a large potential for translation into clinical application, yielding important insights for the development of treatment options specifically tailored to patients with quiescent IBD targeting maladaptive interoceptive fear and abnormal interoception.

## Supplementary information

2023MP00134RR_Supplement

## Data Availability

All fMRI data analyzed for the current study are available in the neurovault repository (https://neurovault.org/collections/VYIHSMNY/). Additional data are available from the corresponding author upon reasonable request.

## References

[1] Harrison NA, Brydon L, Walker C, Gray MA, Steptoe A, Dolan RJ, et al. Neural origins of human sickness in interoceptive responses to inflammation. Biol Psychiatry. 2009;66:415–22. 19409533 10.1016/j.biopsych.2009.03.007 PMC2885492

[2] Quadt L, Critchley HD, Garfinkel SN. The neurobiology of interoception in health and disease. Ann N Y Acad Sci. 2018;1428:112–28. 29974959 10.1111/nyas.13915

[3] Lasselin J, Lekander M, Benson S, Schedlowski M, Engler H. Sick for science: experimental endotoxemia as a translational tool to develop and test new therapies for inflammation-associated depression. Mol Psychiatry. 2021;26:3672–83. 32873895 10.1038/s41380-020-00869-2 PMC8550942

[4] Benson S, Labrenz F, Kotulla S, Brotte L, Rödder P, Tebbe B, et al. Amplified gut feelings under inflammation and depressed mood: a randomized fMRI trial on interoceptive pain in healthy volunteers. Brain Behavior Immunity. 2023;112:132–7. 37302437 10.1016/j.bbi.2023.06.005

[5] Davies KA, Cooper E, Voon V, Tibble J, Cercignani M, Harrison NA. Interferon and anti-TNF therapies differentially modulate amygdala reactivity which predicts associated bidirectional changes in depressive symptoms. Mol Psychiatry. 2021;26:5150–60. 32457424 10.1038/s41380-020-0790-9 PMC8589643

[6] Harrison NA, Brydon L, Walker C, Gray MA, Steptoe A, Critchley HD. Inflammation causes mood changes through alterations in subgenual cingulate activity and mesolimbic connectivity. Biol Psychiatry. 2009;66:407–14. 19423079 10.1016/j.biopsych.2009.03.015 PMC2885494

[7] Kraynak TE, Marsland AL, Wager TD, Gianaros PJ. Functional neuroanatomy of peripheral inflammatory physiology: a meta-analysis of human neuroimaging studies. Neurosci Biobahv Rev. 2018;94:76–92. 10.1016/j.neubiorev.2018.07.013 PMC6363360 30067939

[8] Pawlik RJ, Petrakova L, Cueillette A, Krawczyk K, Theysohn N, Elsenbruch S, et al. Inflammation shapes neural processing of interoceptive fear predictors during extinction learning in healthy humans. Brain Behav Immun. 2023;108:328–39. 36535608 10.1016/j.bbi.2022.12.010

[9] Bishop SJ. Neural mechanisms underlying selective attention to threat. Ann N Y Acad Sci. 2008;1129:141–52. 18591476 10.1196/annals.1417.016

[10] Bhatt RR, Gupta A, Labus JS, Liu C, Vora PP, Jean S, et al. A neuropsychosocial signature predicts longitudinal symptom changes in women with irritable bowel syndrome. Mol Psychiatry. 2022;27:1774–91. 34819635 10.1038/s41380-021-01375-9 PMC9095468

[11] Mayer EA, Ryu HJ, Bhatt RR. The neurobiology of irritable bowel syndrome. Mol Psychiatry. 2023;28:1451–65. 36732586 10.1038/s41380-023-01972-w PMC10208985

[12] Wang H, Labus JS, Griffin F, Gupta A, Bhatt RR, Sauk JS, et al. Functional brain rewiring and altered cortical stability in ulcerative colitis. Mol Psychiatry. 2022;27:1792–804. 35046525 10.1038/s41380-021-01421-6 PMC9095465

[13] Bakshi N, Hart AL, Lee MC, Williams ACC, Lackner JM, Norton C, et al. Chronic pain in patients with inflammatory bowel disease. Pain. 2021;162:2466–71. 34534174 10.1097/j.pain.0000000000002304 PMC8442739

[14] Sweeney L, Moss-Morris R, Czuber-Dochan W, Meade L, Chumbley G, Norton C. Systematic review: psychosocial factors associated with pain in inflammatory bowel disease. Aliment Pharmacol Ther. 2018;47:715–29. 29359343 10.1111/apt.14493

[15] Fairbrass KM, Costantino SJ, Gracie DJ, Ford AC. Prevalence of irritable bowel syndrome-type symptoms in patients with inflammatory bowel disease in remission: a systematic review and meta-analysis. Lancet Gastroenterol Hepatol. 2020;5:1053–62. 33010814 10.1016/S2468-1253(20)30300-9

[16] Dempsey E, Abautret-Daly Á, Docherty NG, Medina C, Harkin A. Persistent central inflammation and region specific cellular activation accompany depression- and anxiety-like behaviours during the resolution phase of experimental colitis. Brain Behav Immun. 2019;80:616–32. 31063848 10.1016/j.bbi.2019.05.007

[17] Huang T, Okauchi T, Hu D, Shigeta M, Wu Y, Wada Y, et al. Pain matrix shift in the rat brain following persistent colonic inflammation revealed by voxel-based statistical analysis. Mol Pain. 2019;15:1–3. 10.1177/1744806919891327 PMC6886279 31709891

[18] Matisz CE, Patel M, Hong NS, McDonald RJ, Gruber AJ. Chronic gut inflammation impairs contextual control of fear. Sci Rep. 2022;12:20586. 36446873 10.1038/s41598-022-24901-3 PMC9709066

[19] Salameh E, Meleine M, Gourcerol G, do Rego JC, do Rego JL, Legrand R, et al. Chronic colitis-induced visceral pain is associated with increased anxiety during quiescent phase. Am J Physiol Gastrointest Liver Physiol. 2019;316:G692–G700. 30735453 10.1152/ajpgi.00248.2018

[20] Bao C, Liu P, Liu H, Jin X, Calhoun VD, Wu L, et al. Different brain responses to electro-acupuncture and moxibustion treatment in patients with Crohn’s disease. Sci Rep. 2016;6:36636. 27857211 10.1038/srep36636 PMC5114555

[21] Li L, Ma J, Xu JG, Zheng YL, Xie Q, Rong L, et al. Brain functional changes in patients with Crohn’s disease: A resting-state fMRI study. Brain Behav. 2021;11:e2243. 34124857 10.1002/brb3.2243 PMC8413760

[22] Zhang S, Chen F, Wu J, Liu C, Yang G, Piao R, et al. Altered structural covariance and functional connectivity of the insula in patients with Crohn’s disease. Quant Imaging Med Surg. 2022;12:1020–36. 35111602 10.21037/qims-21-509 PMC8739134

[23] Öhlmann H, Lanters LR, Theysohn N, Langhorst J, Engler H, Icenhour A, et al. Distinct alterations in central pain processing of visceral and somatic pain in quiescent ulcerative colitis compared to irritable bowel syndrome and health. J Crohns Colitis. 2023;17:1639–51. 37161902 10.1093/ecco-jcc/jjad080 PMC10637045

[24] Rubio A, Pellissier S, Van Oudenhove L, Ly HG, Dupont P, Tack J, et al. Brain responses to uncertainty about upcoming rectal discomfort in quiescent Crohn’s disease - a fMRI study. Neurogastroenterol Motil. 2016;28:1419–32. 27132547 10.1111/nmo.12844

[25] Keefer L, Ballou SK, Drossman DA, Ringstrom G, Elsenbruch S, Ljotsson B. A Rome working team report on brain-gut behavior therapies for disorders of gut-brain interaction. Gastroenterology. 2021;162:300–15. 34529986 10.1053/j.gastro.2021.09.015

[26] Vlaeyen JWS. Learning to predict and control harmful events. Pain. 2015;156:S86–S93. 25789440 10.1097/j.pain.0000000000000107

[27] Icenhour A, Langhorst J, Benson S, Schlamann M, Hampel S, Engler H, et al. Neural circuitry of abdominal pain-related fear learning and reinstatement in irritable bowel syndrome. Neurogastroenterol Motil. 2015;27:114–27. 25557224 10.1111/nmo.12489

[28] Koenen LR, Pawlik RJ, Icenhour A, Petrakova L, Forkmann K, Theysohn N, et al. Associative learning and extinction of conditioned threat predictors across sensory modalities. Commun Biol. 2021;4:1–17. 33976383 10.1038/s42003-021-02008-1 PMC8113515

[29] 0Staudacher HM, Mikocka-Walus A, Ford AC. Common mental disorders in irritable bowel syndrome: pathophysiology, management, and considerations for future randomised controlled trials. Lancet Gastroenterol Hepatol. 2021;6:401–10. 33587890 10.1016/S2468-1253(20)30363-0

[30] Kucharzik T, Koletzko S, Kannengießer K, Dignaß A. Ulcerative colitis - diagnostic and therapeutic algorithms. Dtsch Arztebl Int. 2020;117:564–73. 33148393 10.3238/arztebl.2020.0564 PMC8171548

[31] Rachmilewitz D. Coated mesalazine (5-aminosalicylic acid) versus sulphasalazine in the treatment of active ulcerative colitis: a randomised trial. BMJ. 1989;298:82–6. 2563951 10.1136/bmj.298.6666.82 PMC1835436

[32] Ferretti F, Cannatelli R, Monico MC, Maconi G, Ardizzone S. An update on current pharmacotherapeutic options for the treatment of ulcerative colitis. J Clin Med. 2022;11:2302. 10.3390/jcm11092302 PMC9104748 35566428

[33] Actis GC, Pellicano R. Inflammatory bowel disease: efficient remission maintenance is crucial for cost containment. World J Gastrointest Pharmacol Ther. 2017;8:114–9. 28533920 10.4292/wjgpt.v8.i2.114 PMC5421109

[34] Lim WC, Wang Y, MacDonald JK, Hanauer S. Aminosalicylates for induction of remission or response in Crohn’s disease. Cochrane Database Syst Rev. 2016;7:CD008870. 27372735 10.1002/14651858.CD008870.pub2 PMC6457996

[35] Lacy BE, Mearin F, Chang L, Chey WD, Lembo AJ, Simren M, et al. Bowel disorders. Gastroenterology. 2016;150:1393–407. 10.1053/j.gastro.2016.02.031 27144627

[36] Herrmann-Lingen C, Buss U, Snaith RP. Hospital anxiety and depression scale - German Version. Bern: Huber; 2005.

[37] Gierthmuhlen J, Enax-Krumova EK, Attal N, Bouhassira D, Cruccu G, Finnerup NB, et al. Who is healthy? Aspects to consider when including healthy volunteers in QST-based studies-a consensus statement by the EUROPAIN and NEUROPAIN consortia. Pain. 2015;156:2203–11. 26075963 10.1097/j.pain.0000000000000227

[38] Lacourt TE, Houtveen JH, Doornen LJP, Benson S, Grigoleit JS, Cesko E, et al. Biological and psychological predictors of visceral pain sensitivity in healthy premenopausal women. Eur J Pain. 2014;18:567–74. 24027228 10.1002/j.1532-2149.2013.00397.x

[39] Radbruch L, Loick G, Kiencke P, Lindena G, Sabatowski R, Grond S, et al. Validation of the german version of the brief pain inventory. J Pain Symptom Manage. 1999;18:180–7. 10517039 10.1016/s0885-3924(99)00064-0

[40] Schulz P, Schlotz W. The Trier inventory for the assessment of chronic stress (TICS): scale construction, statistical testing, and validation of the scale work overload. Diagnostica. 1999;45:8–19.

[41] Mavroudis G, Strid H, Jonefjall B, Simren M. Visceral hypersensitivity is together with psychological distress and female gender associated with severity of IBS-like symptoms in quiescent ulcerative colitis. Neurogastroenterol Motil. 2021;33:e13998. 33034406 10.1111/nmo.13998

[42] Roberts C, Albusoda A, Farmer AD, Aziz Q. Rectal hypersensitivity in inflammatory bowel disease: a systematic review and meta-analysis. Crohn’s Colitis 360. 2021;3:otab041. 36776657 10.1093/crocol/otab041 PMC9802320

[43] Kattoor J, Gizewski ER, Kotsis V, Benson S, Gramsch C, Theysohn N, et al. Fear conditioning in an abdominal pain model: neural responses during associative learning and extinction in healthy subjects. PLoS ONE. 2013;8:e51149. 23468832 10.1371/journal.pone.0051149 PMC3582635

[44] Labrenz F, Icenhour A, Benson S, Elsenbruch S. Contingency awareness shapes acquisition and extinction of emotional responses in a conditioning model of pain-related fear. Front Behav Neurosci. 2015;9:1–9. 26640433 10.3389/fnbeh.2015.00318 PMC4661267

[45] Labrenz F, Icenhour A, Schlamann M, Forsting M, Bingel U, Elsenbruch S. From Pavlov to pain: How predictability affects the anticipation and processing of visceral pain in a fear conditioning paradigm. NeuroImage. 2016;130:104–14. 26854560 10.1016/j.neuroimage.2016.01.064

[46] Öhlmann H, Koenen LR, Labrenz F, Engler H, Theysohn N, Langhorst J, et al. Altered brain structure in chronic visceral pain: specific differences in gray matter volume and associations with visceral symptoms and chronic stress. Front Neurol. 2021;12:733035. 34744973 10.3389/fneur.2021.733035 PMC8564184

[47] Fullana MA, Albajes-Eizagirre A, Soriano-Mas C, Vervliet B, Cardoner N, Benet O, et al. Fear extinction in the human brain: A meta-analysis of fMRI studies in healthy participants. Neurosci Biobehav Rev. 2018;88:16–25. 29530516 10.1016/j.neubiorev.2018.03.002

[48] Fullana MA, Harrison BJ, Soriano-Mas C, Vervliet B, Cardoner N, Avila-Parcet A, et al. Neural signatures of human fear conditioning: an updated and extended meta-analysis of fMRI studies. Mol Psychiatry. 2015;21:500–8. 26122585 10.1038/mp.2015.88

[49] Pico-Perez M, Alemany-Navarro M, Dunsmoor JE, Radua J, Albajes-Eizagirre A, Vervliet B, et al. Common and distinct neural correlates of fear extinction and cognitive reappraisal: a meta-analysis of fMRI studies. Neurosci Biobehav Rev. 2019;104:102–15. 31278951 10.1016/j.neubiorev.2019.06.029

[50] Büchel C, Dolan RJ. Classical fear conditioning in functional neuroimaging. Curr Opinn Neurobiol. 2000;10:219–23. 10.1016/s0959-4388(00)00078-7 10753800

[51] LeDoux JE. Coming to terms with fear. Proc Natl Acad Sci USA. 2014;111:2871–8. 24501122 10.1073/pnas.1400335111 PMC3939902

[52] Harrison NA. Commentary on the 2016 named series: neuroimaging, inflammation and behavior. Brain Behav Immun. 2016;58:48–51. 27531190 10.1016/j.bbi.2016.08.010

[53] Agostini A, Filippini N, Benuzzi F, Bertani A, Scarcelli A, Leoni C, et al. Functional magnetic resonance imaging study reveals differences in the habituation to psychological stress in patients with Crohn’s disease versus healthy controls. J Behav Med. 2013;36:477–87. 22752251 10.1007/s10865-012-9441-1

[54] Agostini A, Filippini N, Cevolani D, Agati R, Leoni C, Tambasco R, et al. Brain functional changes in patients with ulcerative colitis: a functional magnetic resonance imaging study on emotional processing. Inflammatory Bowel Diseases. 2011;17:1769–77. 21744432 10.1002/ibd.21549

[55] Kong E, Monje FJ, Hirsch J, Pollak DD. Learning not to fear: neural correlates of learned safety. Neuropsychopharmacology. 2014;39:515–27. 23963118 10.1038/npp.2013.191 PMC3895233

[56] Laing PAF, Steward T, Davey CG, Felmingham KL, Fullana MA, Vervliet B, et al. Cortico-striatal activity characterizes human safety learning via Pavlovian conditioned inhibition. J Neurosci. 2022;42:5047–57. 35577553 10.1523/JNEUROSCI.2181-21.2022 PMC9233447

[57] Jarcho JM, Feier NA, Bert A, Labus JA, Lee M, Stains J, et al. Diminished neurokinin-1 receptor availability in patients with two forms of chronic visceral pain. Pain. 2013;154:987–96. 23582152 10.1016/j.pain.2013.02.026 PMC4294187

[58] Mayer EA, Berman S, Suyenobu B, Labus J, Mandelkern MA, Naliboff BD, et al. Differences in brain responses to visceral pain between patients with irritable bowel syndrome and ulcerative colitis. Pain. 2005;115:398–409. 15911167 10.1016/j.pain.2005.03.023

[59] Schmid J, Langhorst J, Gaß F, Theysohn N, Benson S, Engler H, et al. Placebo analgesia in patients with functional and organic abdominal pain: a fMRI study in IBS, UC and healthy volunteers. Gut. 2015;64:418–27. 24833636 10.1136/gutjnl-2013-306648

[60] Quigley EM. Overlapping irritable bowel syndrome and inflammatory bowel disease: less to this than meets the eye? Ther Adv Gastroenterol. 2016;9:199–212. 10.1177/1756283X15621230 PMC4749858 26929782

[61] Spiller R, Major G. IBS and IBD — separate entities or on a spectrum? Nat Rev Gastroenterol Hepatol. 2016;13:613–21. 27667579 10.1038/nrgastro.2016.141

[62] Jenewein J, Moergeli H, Sprott H, Honegger D, Brunner L, Ettlin D, et al. Fear-learning deficits in subjects with fibromyalgia syndrome? Eur J Pain. 2013;17:1374–84. 23468076 10.1002/j.1532-2149.2013.00300.x PMC3929307

[63] Sandström A, Ellerbrock I, Lofgren M, Altawil R, Bileviciute-Ljungar I, Lampa J, et al. Distinct aberrations in cerebral pain processing differentiating patients with fibromyalgia from patients with rheumatoid arthritis. Pain. 2022;163:538–47. 34224497 10.1097/j.pain.0000000000002387 PMC8832547

[64] Benson S, Rebernik L, Pastoors D, Brinkhoff A, Wegner A, Elsenbruch S, et al. Impact of acute inflammation on the extinction of aversive gut memories. Brain Behav Immun. 2020;88:294–301. 32531428 10.1016/j.bbi.2020.06.009

[65] Gracie DJ, Hamlin PJ, Ford AC. The influence of the brain – gut axis in inflammatory bowel disease and possible implications for treatment. Lancet Gastroenterol Hepatol. 2019;4:632–42. 31122802 10.1016/S2468-1253(19)30089-5

[66] Koenen LR, Icenhour A, Forkmann K, Pasler A, Theysohn N, Forsting M, et al. Greater fear of visceral pain contributes to differences between visceral and somatic pain in healthy women. Pain. 2017;158:1599–608. 28426553 10.1097/j.pain.0000000000000924

[67] Strigo IA, Bushnell MC, Boivin M, Duncan GH. Psychophysical analysis of visceral and cutaneous pain in human subjects. Pain. 2002;97:235–46. 12044620 10.1016/S0304-3959(02)00023-4

[68] Lacheze C, Coelho A-M, Fioramonti J, Bueno L. Influence of trimebutine on inflammation- and stress-induced hyperalgesia to rectal distension in rats. J Pharmacy Pharmacol. 1998;50:921–8. 10.1111/j.2042-7158.1998.tb04009.x 9751458

[69] Bras JP, Guillot de Suduiraut I, Zanoletti O, Monari S, Meijer M, Grosse J, et al. Stress-induced depressive-like behavior in male rats is associated with microglial activation and inflammation dysregulation in the hippocampus in adulthood. Brain Behav Immun. 2022;99:397–408. 34793941 10.1016/j.bbi.2021.10.018

[70] Labanski A, Langhorst J, Engler H, Elsenbruch S. Stress and the brain-gut axis in functional and chronic-infl ammatory gastrointestinal diseases: a transdisciplinary challenge. Psychoneuroendocrinology. 2020;111:104501. 31715444 10.1016/j.psyneuen.2019.104501

[71] Reber SO. Stress and animal models of inflammatory bowel disease-an update on the role of the hypothalamo-pituitary-adrenal axis. Psychoneuroendocrinology. 2012;37:1–19. 21741177 10.1016/j.psyneuen.2011.05.014

[72] Straub RH, Herfarth H, Falk W, Andus T, Schölmerich J. Uncoupling of the sympathetic nervous system and the hypothalamic-pituitary-adrenal axis in inflammatory bowel disease? J Neuroimmunol. 2002;126:116–25. 12020963 10.1016/s0165-5728(02)00047-4

[73] Icenhour A, Petrakova L, Hazzan N, Theysohn N, Merz CJ, Elsenbruch S. When gut feelings teach the brain to fear pain: Context-dependent activation of the central fear network in a novel interoceptive conditioning paradigm. Neuroimage. 2021;238:118229. 34082119 10.1016/j.neuroimage.2021.118229

[74] Wils P, Caron B, D’Amico F, Danese S, Peyrin-Biroulet L. Abdominal pain in inflammatory bowel diseases: a clinical challenge. J Clin Med. 2022;11:4269. 10.3390/jcm11154269 PMC9331632 35893357

[75] Robertson N, Gunn S, Piper R. Psychological and social factors associated with pain in inflammatory bowel disease : a systematic literature review of the evidence in adult and pediatric studies. Crohn’s Colitis. 2019;1:1–19.

[76] Schirbel A, Reichert A, Roll S, Baumgart DC, Buning C, Wittig B, et al. Impact of pain on health-related quality of life in patients with inflammatory bowel disease. World J Gastroenterol. 2010;16:3168–77. 20593502 10.3748/wjg.v16.i25.3168 PMC2896754

[77] Hetterich L, Stengel A. Psychotherapeutic interventions in irritable bowel syndrome. Front Psychiatry. 2020;11:286. 32425821 10.3389/fpsyt.2020.00286 PMC7205029

[78] Peppas S, Pansieri C, Piovani D, Danese S, Peyrin-Biroulet L, Tsantes AG, et al. The brain-gut axis: psychological functioning and inflammatory bowel diseases. J Clin Med. 2021;10:377. 10.3390/jcm10030377 PMC7863941 33498197

