# Supplementary material for: Disease-specific alterations in central fear network engagement during acquisition and extinction of conditioned interoceptive fear in inflammatory bowel disease
Source: Mol Psychiatry. 2024 May 27;29(11):3527–36. doi: 10.1038/s41380-024-02612-7 (PMC11541002; doi:10.1038/s41380-024-02612-7)
Supplement: Supplementary file 1 — 2023MP00134RR_Supplement [file 41380_2024_2612_MOESM1_ESM.docx]

**SUPPLEMENTARY MATERIALS**

**Disease-specific alterations in central fear network engagement during acquisition and extinction of conditioned interoceptive fear in inflammatory bowel disease**

Laura R. Lanters^1^ (Ph.D.), Hanna Öhlmann^2^ (Ph.D.), Jost Langhorst^3,4^ (MD), Nina Theysohn^5^ (MD), Harald Engler^6^ (Ph.D.), Adriane Icenhour^2,#^ (Ph.D.), Sigrid Elsenbruch^1,2,#^ (Ph.D.)

^#^These authors contributed equally to this work

^1^ Department of Neurology, Center for Translational Neuro- and Behavioral Sciences, University Hospital Essen, University of Duisburg-Essen, Germany

^2^ Department of Medical Psychology and Medical Sociology, Ruhr University Bochum, Germany

^3^ Department for Internal and Integrative Medicine, Sozialstiftung Bamberg, Germany

^4^ Department for Integrative Medicine, Medical Faculty, University of Duisburg-Essen, Germany

^5^ Institute of Diagnostic and Interventional Radiology and Neuroradiology, University Hospital Essen, University of Duisburg-Essen, Germany

^6^ Institute of Medical Psychology and Behavioral Immunobiology, Center for Translational Neuro- and Behavioral Sciences, University Hospital Essen, University of Duisburg-Essen, Germany

**1 SUPPLEMENTAL METHODS**

**S1.1 General exclusion criteria**

The highly standardized recruitment and screening process consisted of a structured telephone screening, a personal interview, a medical examination, and completion of psychosocial and clinical symptom questionnaires. General exclusion criteria were the usual contraindications for magnetic resonance imaging (MRI; e.g., claustrophobia, ferromagnetic implants), structural brain abnormalities (ruled out by a licensed neuroradiologist, author N.T.), and left-handedness assessed with a validated questionnaire (1). As rectal distensions were used as painful interoceptive US herein, perianal tissue damage (e.g., hemorrhoids, fissures) was excluded by digital rectal examination. Given well-documented sex differences in both pain perception and processing (2) as well as in aversive learning and memory processes (3), only women were recruited for all groups. Pregnancy was ruled out using a commercially available urinary pregnancy test (Biorepair GmbH, Sinsheim, Germany) on the day of the study.

**S1.2 Experimental procedures accomplished prior to fear conditioning**

As previously described in detail for implementation in healthy volunteers (4), procedures accomplished before fear conditioning consisted of the determination of individual pain thresholds for the visceral and the somatic pain modality, respectively, followed by the titration and matching of individual pain stimulation intensities and a pain habituation phase [(results from the same patient cohorts reported in (5)]. Visceral pain stimuli were implemented with pressure-controlled rectal balloon distensions, delivered with a barostat system (modified ISOBAR 3 device, G & J Electronics, Toronto, ON, Canada), as a clinically relevant and widely used visceral pain model (details e.g. in (4-6). For the somatic pain modality, thermal cutaneous pain stimuli were applied on the left ventral forearm using a thermode (PATHWAY model CHEPS; Medoc Ltd. Advanced Medical Systems, Ramat Yishai, Israel). For implementation as US, based on pain thresholds, distension pressures and temperatures were individually titrated to achieve perceived pain intensities within a predefined target range of 60-80 mm on a visual analog scale (VAS, 0-100 mm, ends labelled “not painful at all” and “extremely painful”) and matched for perceived pain intensity. Note that implementation of individually titrated US stimulus intensities during the acquisition phase controls for interindividual variability in pain sensitivity and minimizes possible effects of condition-specific hypersensitivity (e.g., as has been observed in IBS or in some IBD patients; 7-9).

Immediately prior to conditioning, a pain habituation phase was accomplished. This facilitated a familiarization of participants with the scanning environment and allowed us to verify that individually titrated stimulus intensities induced adequate pain levels, as a crucial basis for implementation of effective US during conditioning. To this end, five painful rectal distensions and five thermal cutaneous pain stimuli were presented in randomized order and without predictive cues. In case pain ratings indicated a deviation from the target range, stimulus intensities were adjusted before conditioning. Note that for safety reasons, the maximum pressure applied was limited to 60 mmHg and the maximum temperature applied was limited to 50 °C.

**S1.3 Expanded methods brain imaging**

All MR images were acquired using a whole-body 3 Tesla scanner (Skyra, Siemens Healthcare, Erlangen, Germany) equipped with a 32-channel head coil. A T1-weighted 3D-magnetization prepared rapid gradient echo (MPRAGE) sequence was used to assess structural images (repetition time (TR) 1900ms, echo time (TE) 2.13ms, flip angle 9°, field of view (FOV) 239 x 239mm^2^, 192 slices, slice thickness 0.9mm, voxel size 0.9 x 0.9 x 0.9mm^3^, matrix 256 x 256mm^2^, Generalized Partially Parallel Acquisitions (GRAPPA) r=2). Structural MR-images were used in a larger voxel-based morphometry analysis to examine structural brain alterations in UC and IBS (10). For functional imaging, a single-shot echo-planar imaging (EPI) sequence was used (TR 2300ms, TE 28.0ms, flip angle 90°, FOV 220 x 220 mm^2^, matrix 94 x 94 mm^2^, GRAPPA r = 2 with 38 transversal slices angulated in the direction of the corpus callosum, slice thickness of 3mm, slice gap 0.6mm, voxel size 2.3 x 2.3 x 3.0mm) to measure event-related blood oxygen level-dependent (BOLD) responses.

Functional images were analyzed with SPM software (SPM12, Wellcome Trust Centre for Neuroimaging, UCL, London, UK) implemented in Matlab (R2021a, Mathworks Inc., Sherborn, MA, USA). A standard realignment procedure was performed as implemented in SPM12 for the estimation of six parameters for translation (x, y, z) and for rotation (pitch, roll, yaw) to describe the rigid body transformation between each image and a reference image. Functional images were co-registered to individual T1-weighted structural images used as reference images, with the origin set to the anterior commissure, and normalized to Montreal Neurological Institute (MNI) space using a standardized International Consortium for Brain Mapping (ICBM) template for European brains and smoothed using an isotropic Gaussian kernel of 8 mm. A temporal high-pass filter with a cut-off set at 128 s was implemented to correct for low frequency drifts, and serial autocorrelations were taken into consideration by means of an autoregressive model first-order correction.

First- and second-level analyses are described in full detail in the main manuscript.

All analyses focused on *a priori* defined regions of interest (ROI) of the fear and extinction networks (11-13), including amygdala, hippocampus, ventromedial prefrontal cortex (vmPFC), anterior insula (aINS), anterior cingulate cortex (ACC), thalamus, and basal ganglia (putamen, pallidum, caudate). Segmentation of aINS and ACC was accomplished with masks based on the previous literature (14) within the borders of the Wake Forest University (WFU) Pick Atlas. All ROI analyses were carried out using unilateral anatomical templates constructed from the WFU Pick Atlas (Version 2.5.2), as implemented in SPM12. For all reported ROI-analyses, familywise-error (FWE) correction for multiple testing was used with statistical significance set at *p_FWE_* < 0.05 and a minimal cluster size (*k_E_*) of 3, and coordinates refer to the MNI space. Plots were prepared using the software MRIcroGL (version 1.2.20210317; 15). Parameter estimates from peak-voxels identified in significant group comparisons were extracted from the respective ROI to visualize the direction of observed effects with the ggplot2 package (16) using R (Version 4.1.1; R Core Team, Vienna, Austria) in RStudio (Version 2021.09.0+351; RStudio, Boston, MA), and to accomplish exploratory correlational analyses.

**S1.4 References cited within supplemental methods sections S1.1 – S1.3**

1. Oldfield RC. The assessment and analysis of handedness: the edinburgh inventory. Neuropsychologica. 1971;9:97-113.

2. Racine M, Tousignant-Laflamme Y, Kloda LA, Dion D, Dupuis G, Choiniere M. A systematic literature review of 10 years of research on sex/gender and pain perception - part 2: do biopsychosocial factors alter pain sensitivity differently in women and men? Pain. 2012;153(3):619-35.

3. Day HLL, Stevenson CW. The neurobiological basis of sex differences in learned fear and its inhibition. Eur J Neurosci. 2020;52(1):2466-86.

4. Koenen LR, Pawlik RJ, Icenhour A, Petrakova L, Forkmann K, Theysohn N, et al. Associative learning and extinction of conditioned threat predictors across sensory modalities. Commun Biol. 2021;4:1-17.

5. Öhlmann H, Lanters LR, Theysohn N, Langhorst J, Engler H, Icenhour A, et al. Distinct alterations in central pain processing of visceral and somatic pain in quiescent ulcerative colitis compared to irritable bowel syndrome and health. J Crohns Colitis. 2023.

6. Koenen LR, Icenhour A, Forkmann K, Pasler A, Theysohn N, Forsting M, et al. Greater fear of visceral pain contributes to differences between visceral and somatic pain in healthy women. Pain. 2017;158(8):1599-608.

7. Mavroudis G, Strid H, Jonefjall B, Simren M. Visceral hypersensitivity is together with psychological distress and female gender associated with severity of IBS-like symptoms in quiescent ulcerative colitis. Neurogastroenterol Motil. 2021;33(3):e13998.

8. Mayer EA, Ryu HJ, Bhatt RR. The neurobiology of irritable bowel syndrome. Mol Psychiatry. 2023;28:1451-65.

9. Roberts C, Albusoda A, Farmer AD, Aziz Q. Rectal hypersensitivity in inflammatory bowel disease: a systematic review and meta-analysis. Crohn's & Colitis 360. 2021;3(3).

10. Öhlmann H, Koenen LR, Labrenz F, Engler H, Theysohn N, Langhorst J, et al. Altered brain structure in chronic visceral pain: specific differences in gray matter volume and associations with visceral symptoms and chronic stress. Front Neurol. 2021;12:733035.

11. Fullana MA, Albajes-Eizagirre A, Soriano-Mas C, Vervliet B, Cardoner N, Benet O, et al. Fear extinction in the human brain: A meta-analysis of fMRI studies in healthy participants. Neurosci Biobehav Rev. 2018;88:16-25.

12. Fullana MA, Harrison BJ, Soriano-Mas C, Vervliet B, Cardoner N, Avila-Parcet A, et al. Neural signatures of human fear conditioning: an updated and extended meta-analysis of fMRI studies. Mol Psychiatry. 2015;21(4):500-8.

13. Pico-Perez M, Alemany-Navarro M, Dunsmoor JE, Radua J, Albajes-Eizagirre A, Vervliet B, et al. Common and distinct neural correlates of fear extinction and cognitive reappraisal: A meta-analysis of fMRI studies. Neurosci Biobehav Rev. 2019;104:102-15.

14. Deen B, Pitskel NB, Pelphrey KA. Three systems of insular functional connectivity identified with cluster analysis. Cereb Cortex. 2011;21(7):1498-506.

15. Rorden C, Brett M. Stereotaxic display of brain lesions. Behav Neurol. 2000;12:191-200.

16. Wickham H. Ggplot2: elegant graphics for data analysis. New York: Springer-Verlag; 2016.

**2 SUPPLEMENTAL RESULTS**

**S2.1 Assessment of medication effects on group differences observed between UC and comparison groups**

To examine whether medications taken by UC patients impacted on the observed group differences between UC and comparison groups in central fear network engagement during the acquisition and extinction of interoceptive fear, we accomplished a supplemental analysis. Here, we excluded the UC patients who were at the time of our study treated with systemically acting anti-inflammatory medications (i.e., *N*=2 patients on a TNF-α blocker and *N*=2 patients on azathioprine) and accomplished the same group comparisons as reported in the main manuscript (compare to Table 2). Results revealed that the exclusion of *N*=4 patients from the UC cohort did not substantially alter results of BOLD-analyses on differential activation induced by conditioned predictors of visceral pain (i.e., ΔCS_VISC_): Albeit with smaller cluster sizes, all clusters for which alterations in UC vs. HC were reported for the late acquisition phase remained significant (left amygdala: *x* = -28, *y* = -6, *z* = -18, *t* = 3.31, *p*_FWE_ = .027, *k*_E_ = 8; left hippocampus: *x* = -26, *y* = -8, *z* = -22, *t* = 3.70, *p*_FWE_ = .039, *k*_E_ = 1; right putamen: *x* = 32, *y* = 10, *z* = -2, *t* = 3.68, *p*_FWE_ = .041, *k*_E_ = 1). For the comparison of UC and IBS, clusters that emerged during late acquisition within dorsal aINS and putamen did not reach significance, while group differences were still observed within left amygdala (*x* = -28, *y* = 0, *z* = -28, *t* = 3.28, *p*_FWE_ = .034, *k*_E_ = 2). During fear extinction, all reported clusters remained significant (left amygdala: *x* = -22, *y* = -6, *z* = -12, *t* = 3.56, *p*_FWE_ = .010, *k*_E_ = 26; left ventral aINS: *x* = -40, y = 4, *z* = -10, *t* = 3.92, *p*_FWE_ = .015, *k*_E_ = 6; right thalamus: *x* = 4, *y* = -24, *z* = 4, *t* = 3.63, *p*_FWE_ = .027, *k*_E_ = 3).

In addition to this supplemental statistical analysis, we explored response patterns of differential neural activation during acquisition and extinction, visualized as parameter estimates, including only UC who were completely untreated (i.e., who were taking neither systemically acting anti-inflammatory drugs nor were treated with locally acting medications). Since the number of completely untreated patients in our sample is very small (N=5), we abstained from statistical analyses but inspected visualized parameter estimates. In supplemental Figures S1 (acquisition phase) and S2 (extinction phase), we provide plots allowing a visual comparison of parameter estimates as reported in the main manuscript based on group comparisons in the full UC cohort (upper rows, identical with main figures 2 and 3, respectively) versus group comparisons in the untreated UC subgroup (lower rows). The response patterns show comparable responses for almost all ROI, together indicating that medication status is unlikely to be a causal factor underlying the group differences we report on in the main manuscript.

**Acquisition Phase**


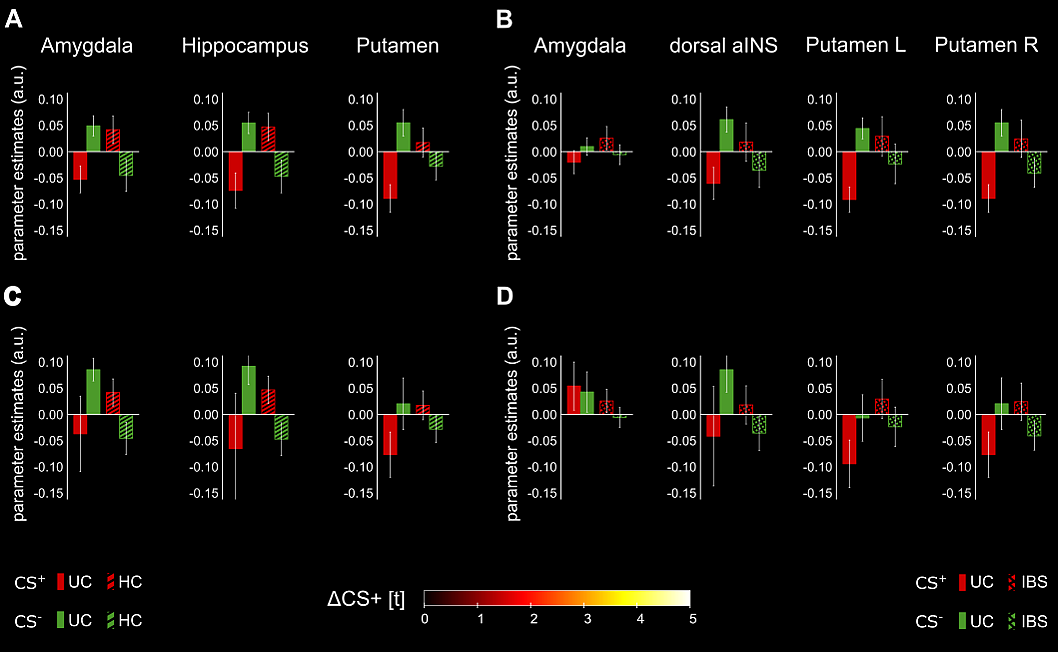


Figure S1. Parameter estimates extracted from analyses on differential blood oxygen level dependent (BOLD) responses induced by conditioned predictors of interoceptive pain (i.e., ΔCS^+^_VISC_) in the late acquisition phase as shown in the main manuscript (compare to figure 2) in UC vs. HC (A) and UC vs. IBS (B), here with the addition of the same response patterns only with the untreated subgroup of UC patients compared to HC (C) and IBS (D). Note that given small and different sample sizes, results are only provided for visual inspection, and no statistical analyses were accomplished for added results shown in C and D.

**Extinction Phase**


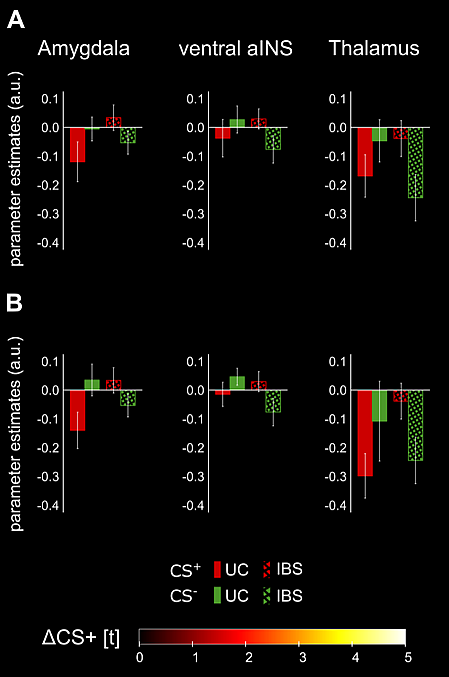
Figure S2. Parameter estimates extracted from analyses on differential blood oxygen level dependent (BOLD) responses induced by conditioned predictors of interoceptive pain (i.e., ΔCS^+^_VISC_) in the extinction phase comparing UC vs. IBS as shown in the manuscript (A, compare to main Figure 3) here additionally shown only for the untreated subgroup of UC patients vs. IBS (B). Note that given small and different sample sizes, results are only provided for visual inspection, and no statistical analyses were accomplished for added results shown in B.

**S2.2 Analyses with overall psychological distress (HADS total score) as covariate**

In light of group differences observed in psychiatric comorbidity, quantified with the Hospital Anxiety and Depression Scale (HADS), supplemental analyses of covariance (ANCOVA) with HADS total score as a covariate were accomplished. When compared to results computed without HADS total score as a covariate (i.e., ANOVA as reported in the main manuscript), ANCOVA did not appreciably change statistical results reported for the analyses of perceived visceral pain intensity (i.e., US_VISC_ ratings; sign. main effect of time: *F*(2,128) = 3.86, *p* = .024, *η_p_^2^* = .06) or for visceral pain-induced neural activation during fear acquisition (i.e., US_VISC_-related BOLD responses; altered engagement of right amygdala in early acquisition in UC compared to HC: x = 20, y = -2, z = -16, *t* = 3.44, *p_FWE_* = .021, *k_E_* = 4). Similarly, behavioral results on differential changes in cue valence (i.e., ΔCS_VISC_ ratings), remained virtually unchanged, with significant main effects of time for the acquisition (*F*(1.54,98.60) = 5.74, *p* = .008, *η_p_^2^* = .08) and extinction phases (*F*(1,64) = 6.12, *p* = .016, *η_p_^2^* = .09). On the other hand, regarding differential activation induced by conditioned predictors of visceral pain (i.e., ΔCS_VISC_-related BOLD responses), after inclusion of HADS total score as additional covariate of no interest in the two-sided independent samples t-tests (UC >/< HC and UC >/< IBS; compare to Table 2), clusters reflecting significant group differences during acquisition and extinction failed to reach suprathreshold levels after rigorous small volume correction.

**S2.3 Acquisition and extinction of exteroceptive fear responses to CS^+^_SOM_**

Analyses reported in the main manuscript focus on fear network reactivity during the acquisition of conditioned interoceptive fear of visceral pain as US in UC compared to HC and IBS as a patient comparison group. However, the translational fear conditioning paradigm also contained thermal cutaneous pain stimuli as an additional exteroceptive (somatic) US (for details on methods, see S1.2), providing the opportunity to explore the specificity of group differences reported for interoceptive (i.e., visceral pain-related) fear to pain modality. To this end, we also provide results of selected supplemented analyses of conditioned exteroceptive fear of somatic pain as US. For analysis of BOLD responses, we applied the identical analysis strategy as described in the main manuscript (i.e., here now entering CS^+^_SOM_ and US_SOM_ as regressors of interest and CS^+^_VISC_ und US_VISC_ as nuisance regressors). Similarly, for behavioural analyses, exteroceptive fear learning was explored by computing the same statistical tests for somatic CS and US (i.e., ΔCS_SOM_ and US_SOM_) as for interoceptive fear learning.

BOLD analyses were focused on testing group differences in exteroceptive fear acquisition (i.e., ΔCS_SOM_) within those selected ROIs revealing group differences for interoceptive fear acquisition (i.e., ΔCS_VISC_), i.e., amygdala, hippocampus, putamen, dorsal and ventral aINS, and thalamus (compare to main Table 2). These analyses yielded no significant findings. This lack of group differences at the neural level was not attributable to altered exteroceptive fear learning efficiency and/or magnitude at the behavioral level, as analyses of ΔCS_SOM_ valence revealed similar findings for exteroceptive CS (i.e., a sign. main effect of time: *F*(2,130) = 14.49, *p* < .001, *η_p_^2^* = .18, but no interaction or group effects) as observed for the interoceptive CS.

For the extinction phase, BOLD analyses comparing ΔCS_SOM_-induced neural responses in UC and HC revealed no significant group differences. On the other hand, as for interoceptive (ΔCS_VISC_-induced) neural responses, group differences between UC and IBS patients emerged in differential engagement in responses to ΔCS_SOM_ within left amygdala (*x* = -26, *y* = -2, *z* = -12, *t* = 3.82, *p_FWE_* = .011, *k_E_* = 30), left ventral aINS (*x* = -36, *y* = 18, *z* = -10, *t* = 3.85, *p_FWE_* = .015, *k*_E_ = 13), and right thalamus (*x* = 14, *y* = -6, *z* = 0, *t* = 4.31, *p_FWE_* = .011, *k*_E_ = 6)]. Behavioral analyses on ΔCS_SOM_ valence demonstrated comparable exteroceptive fear extinction across groups (i.e., a sign. main effect of time: *F*(1,65) = 19.77, *p* < .001, *η_p_^2^* = .23 , but no interaction or group effects).
